# Decoupling Alpha Desynchronization from Neural Resource Use: Evidence from Cognitive Load Modulation

**DOI:** 10.3390/neurosci6020032

**Published:** 2025-04-10

**Authors:** Manuel Vázquez-Marrufo, Rocío Caballero-Díaz, Esteban Sarrias-Arrabal, Rubén Martín-Clemente

**Affiliations:** 1 Experimental Psychology Department, Faculty of Psychology, University of Seville, 41018 Seville, Spain; rcaballero2@us.es; 2 Psychology Department, University of Cadiz, 11001 Cádiz, Spain; esteban.sarrias@gm.uca.es; 3 Institute of Biomedical Research Cadiz (INiBICA), 11009 Cádiz, Spain; 4 Signal Processing and Communications Department, Higher Technical School of Engineering, University of Seville, 41092 Seville, Spain; ruben@us.es

**Keywords:** alpha, cognitive load, evoked, induced, go/no go, P3, reaction time

## Abstract

In prior studies, desynchronization of the induced alpha band (non-phase-locked but time-locked) has been observed across various cognitive tasks. Proposed hypotheses for the cognitive role of this alpha decrement include neural activation, an inhibition/timing mechanism, or a reduction in “neural noise”. This study aimed to examine the effect of cognitive load on induced alpha activity using two versions of a go/no-go visual task: a single-target (ST) version with one target and one distractor, and a double-target (DT) version with two targets and two distractors. EEG was recorded from 58 electrodes, and Temporal Spectral Evolution (TSE) was used for time–frequency analysis. Behavioral results revealed faster reaction times in the ST task compared to the DT task. The P3 component displayed delayed latency and reduced amplitude under increased cognitive load, consistent with prior findings. However, the latencies and amplitudes of evoked and induced alpha responses were unaffected by cognitive load. This suggests that increased alpha desynchronization in subjects with cognitive impairment should not be interpreted as enhanced neural resource recruitment due to task difficulty. Instead, it may reflect other mechanisms unrelated to cognitive load differences in task performance.

## 1. Introduction

Previous studies have broadly focused on the effects of cognitive load on behavior and brain activity [1,2,3]. In this sense, various authors have suggested that cognitive load modulates stimulus processing, recruiting different cognitive mechanisms depending on the demands of the tasks [4,5]. Some psychophysiological modulations related to cognitive load have been principally described in event-related potentials (ERPs) either in pre-stimulus [6,7] or in post stimulus time intervals [4,8]. However, there are very few studies that have analyzed EEG frequency modulations related to cognitive load in visual tasks.

Since the 1970s, various techniques termed “time–frequency” have been developed that allow the modulations of spectral EEG bands to be studied with a resolution of milliseconds (ms) [9,10,11,12]. One of these procedures is temporal spectral evolution (TSE) [13,14]. TSE allows obtaining information about phase (evoked) and non-phase (induced) activity [15,16]. Although TSE has scarcely been applied, it has been demonstrated to be good enough to show cognitive mechanisms hidden for traditional “evoked” analysis [17]. In addition, TSE has also been performed in clinical populations, providing interesting results about altered or compensatory mechanisms [18,19,20,21].

In the study by Vázquez-Marrufo et al. [20], the time–frequency analysis of the EEG showed that gamma band activity was associated with the spatial shifting of attentional focus, while alpha band activity appeared to be linked to expectancy mechanisms and the cognitive processing of the target. Specifically, the alpha band showed a post stimulus decrease that was delayed in time and of greater amplitude compared to that observed in healthy subjects. A similar result was found in another sample of patients and in a different type of visual task [21].

A remarkable aspect of alpha modulations is that all the main properties (latency, amplitude or topographical distribution) of both evoked and induced responses are reliable for longitudinal studies [17]. Moreover, the consistency of post stimulus alpha desynchronization has been confirmed across different paradigms [22,23], other frequency methods (power spectral density) [24] or different time–frequency methods (event-related desynchronization or TSE) [11,25,26].

Independent of the type of analysis applied, authors have tried to associate alpha EEG activity with specific sensory and/or cognitive processes. First, the presence of the alpha band was considered an indicator of neural resting [27]. However, some authors proposed later that alpha activity participates in the inhibitory control and timing of sensory processing [25]. Another way to put it is that an increase in synchronization of alpha activity in irrelevant areas for the task is the reflex of an inhibition process [15,28]. On the other hand, other studies have proposed that the desynchronization of alpha activity is related to the decrease in cortical “neural noise” that competes with stimulus processing [17]. This proposal connects, on the one hand, studies based on ERPs that have shown increases in early components P1 and N1, reflecting enhanced sensory gain of task-relevant stimuli [15], with findings from phase and non-phase spectral modulations suggesting that the phase section is, in fact, modulated by changes observed in non-phase activity [17].

One of the possible functional roles for alpha desynchronization that has been proposed is that the amplitude of the decrement could be correlated with the behavioral responses (the higher the decrement, the better the performance). In fact, some studies have described a positive correlation between poorer behavioral performance and a lower desynchronization of alpha activity in different pathologies [20,22]. Based on this assumption, some authors have suggested that when patients are cognitively impaired, an increase in alpha desynchronization is found, which could represent a special effort from the patient to compensate for the impairment, increasing the resources involved during information processing [20,23].

This line of reasoning in studies with pathological populations has been questioned in some cases because it permits the interpretation of both types of results. If a lower decrement of the alpha band is found in the patient group compared to healthy controls, the mechanism indexed by alpha is impaired, but if the decrement is higher, it means more recruitment of neural resources by the patients as a compensatory mechanism. If the hypothesis of “recruitment of neural resources” is correct, it is logical to think that a more demanding task would increase the decrement of the alpha desynchronization (or delay the latency of its valley) compared to an easier task. The present study investigates this possibility using two go/no go tasks with a different cognitive load level, while EEG signals are recorded in a 58-electrode setup in healthy subjects. This experimental setup was inspired by previous studies that have analyzed how the “cognitive load” variable affects different levels (sensory, motor, etc.) of information processing [3,4,7,8].

## 2. Materials and Methods

### 2.1. Participants

A total of 26 individuals (16 female and 10 male) took part in the study and completed the experiment under controlled conditions. Participants ranged in age from 20 to 52 years, with an average age of 37.3 years (SD = 11.3). Among them, two were left-handed.

### 2.2. Cognitive Tasks

Participants completed the experiment while seated in a sound-attenuated room facing an LCD monitor. The stimuli were programmed using E-Prime 2.0 software (Psychology Software Tools, Inc., Pittsburgh, PA, USA). The cognitive tasks followed a go/no-go paradigm where participants had to discriminate between target visual stimuli (50% probability) and interspersed distractors. Cognitive load was manipulated through two task versions: an easier version with one target and one distractor (single target, ST) and a more challenging version with two targets and two distractors (double target, DT).

Throughout both tasks, a fixation cross remained on screen whenever no stimulus was presented to minimize eye movements. In the ST condition, the target stimulus was a red-and-white checkerboard rectangle, while the distractor shared the same size and pattern but used black and white squares. For the DT condition, targets were rectangles with either red-and-white or blue-and-white checkerboard patterns, whereas distractors featured yellow-and-white or green-and-white patterns of identical dimensions. All stimuli were sized to cover a visual angle of 7.98° × 9.42° at a viewing distance of 70 cm. They were presented one at a time, in either the left or right visual field, with the inner edge of the stimulus positioned 4.11° from the screen center, following a pseudorandom sequence. The selection of these stimuli was primarily inspired by previous tasks [29], which demonstrated results for two types of conditions with different cognitive loads. Additionally, we employed stimuli with high contrast and positioning along the horizontal meridian, which enhanced potential effects on the evoked activity of the EEG signal [15]). Participants were instructed to press the left or right mouse button with their corresponding thumb whenever a target appeared in the left or right visual field, respectively, while ignoring distractors. Each stimulus remained on screen for 500 ms, followed by a 1000 ms interstimulus interval (ISI) during which participants could respond. Each version of the task consisted of 140 trials. At the end of the session, reaction times and accuracy percentages were recorded. Participants were encouraged to respond as quickly and accurately as possible throughout the task.

### 2.3. EEG Recording and Analysis

EEG recordings were obtained from 58 Ag/AgCl electrodes placed according to the standard 10–10 system (American Electroencephalographic Society, 1994) [30] and amplified using BrainAmp amplifiers (Brain Products GmbH, Gilching, Germany). During acquisition, the EEG signals were filtered with a bandpass of 0.01 to 100 Hz, digitized at a sampling rate of 500 Hz, and stored using Brain Vision Recorder 1.27.0001 software (Brain Products GmbH, Gilching, Germany). Data were initially referenced online to the linked earlobes and later re-referenced offline to a common average reference. Electrode impedance was kept below 5 kΩ throughout the experiment. Vertical and horizontal electrooculograms (VEOG and HEOG) were recorded using bipolar montages to monitor eye movements. Trials with horizontal eye movements exceeding ±50 µV were excluded from the analysis. Additionally, blink-related artifacts were corrected on scalp electrodes using the algorithm described by [31]. The continuous EEG data were segmented into epochs ranging from −200 ms to 1000 ms relative to target onset (0 ms). A 200 ms pre-stimulus baseline was included in each epoch to reduce potential edge effects introduced by filtering [10].

After the initial preprocessing, the EEG data were further analyzed to extract both evoked and induced activity. First, target stimulus epochs were averaged in the time domain to obtain ERP modulations, with a particular focus on the P3 component across both task conditions. These averaged signals were then filtered within the alpha frequency range (8–13 Hz) using a zero-phase Butterworth filter (48 dB/octave) and rectified to compute the spectrally evoked activity, followed by a baseline correction applied from −100 to 0 ms, as outlined in [16]. To assess induced activity, the Temporal Spectral Evolution (TSE) method was employed. EEG epochs were filtered in the same alpha band parameters, and the resulting signals were rectified and averaged across target trials. A baseline correction within the −100 to 0 ms window was applied. Finally, the evoked spectral activity was subtracted from the TSE output to isolate the induced response, capturing the non-phase-locked component of the activity, as described in [16].

**Figure 1:**
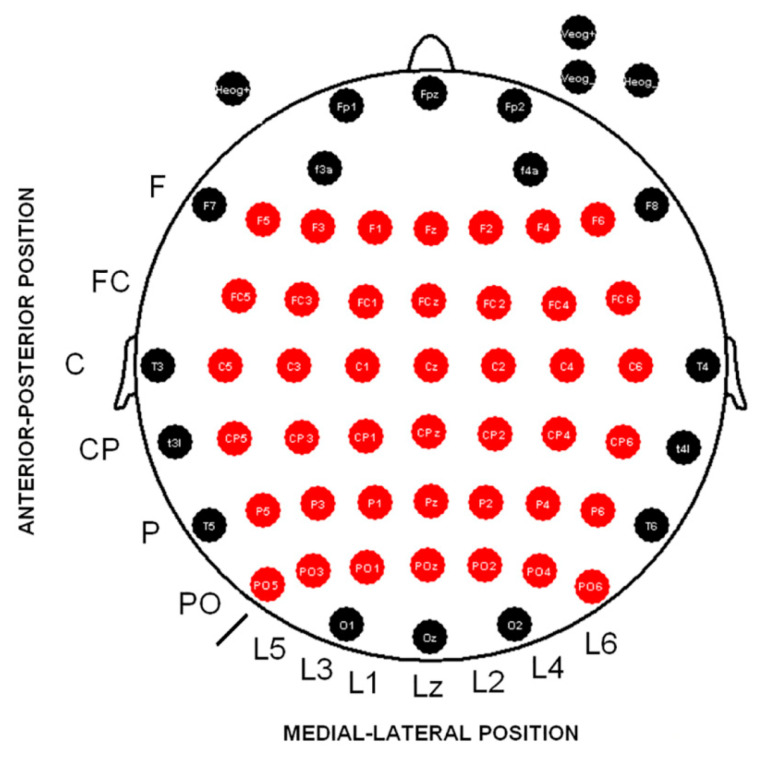
A 6 × 7 electrode matrix for analyses. Fifty-eight scalp derivations are shown. The red electrodes were used to analyze the amplitude differences in all measures between experimental conditions (higher and lower cognitive load). Abbreviations: F (frontal), FC (frontocentral), C (central), CP (central), P (parietal), PO (parietooccipital), L (line), z (zero or midline).

Following the recommendations of two authors [32], latency measurements were taken from the electrode that exhibited the highest amplitude in the grand average of the target conditions. For each participant, the peak latency of the P3 component and both evoked and induced alpha modulations were identified individually, with Pz selected for P3 and PO6 for alpha-related activity. To examine P3 amplitude, the mean value was calculated within the 350–410 ms time window, which captured the peak responses for both task versions (ST and DT). In contrast, the analysis of alpha spectral modulations focused on distinct time intervals depending on the activity type: 140–155 ms for evoked responses and 165–200 ms for induced responses. These windows were selected as they encompassed the maximum amplitude latencies across both tasks. For all analyses involving P3 and alpha modulations, amplitude values were extracted based on the 6 × 7 electrode matrix illustrated in Figure 1.

### 2.4. Phase Analysis for Evoked and Induced Activity

A phase analysis was conducted to rule out any potential influence of evoked activity on the induced response and to confirm that the observed modulation was genuinely non-phase-locked. To accomplish this, the evoked component was estimated by averaging across trials and subsequently subtracting this average from each individual trial, as described in previous studies [33,34]. The data were then filtered within the target frequency range (8–13 Hz) using a zero-phase Butterworth filter (48 dB/octave), and the Hilbert transform was applied to extract the instantaneous phase. Phase measurements for both evoked and induced alpha activity were obtained within the same time windows used for the amplitude analyses.

### 2.5. Statistical Analysis

#### 2.5.1. Behavioral Responses

The Shapiro–Wilk test was applied to evaluate the normality of the reaction time and accuracy data. Since the data did not meet normality assumptions, non-parametric analyses using the Wilcoxon U test were performed to examine significant differences in reaction time and accuracy related to the cognitive load manipulation.

#### 2.5.2. ERP Analysis

The Shapiro–Wilk test was conducted to verify the normality of the P3 latency data. Differences in P3 latency between the ST and DT tasks were examined using a parametric Student’s *t*-test. For the amplitude analysis, an ANOVA was applied, including the following factors: cognitive load (ST vs. DT), anterior–posterior position (frontal, frontocentral, central, centroparietal, parietal, and parietal–posterior), and lateral–medial position (line 5, line 3, line 1, medial, line 2, line 4, and line 6), as illustrated in Figure 1.

#### 2.5.3. Alpha Band Analysis

The Shapiro–Wilk test was applied to assess the normality of alpha latency data for both evoked and induced activities. An ANOVA was then conducted to examine latency differences, considering two factors: cognitive load (ST and DT) and activity type (evoked vs. induced). For amplitude analysis, evoked alpha activity was assessed using ANOVA with the following factors: cognitive load (ST and DT), anterior–posterior position (frontal, frontocentral, central, centroparietal, parietal, and parietal–posterior), and lateral–medial position (line 5, line 3, line 1, medial, line 2, line 4, and line 6). The amplitude of the induced alpha activity was analyzed using the same ANOVA design and factors as those applied for the evoked activity.

In all the statistical analyses described, sphericity was corrected with Greenhouse–Geisser, and a statistically significant result was considered at *p* < 0.05. Post hoc analyses were performed using Bonferroni correction.

## 3. Results

### 3.1. Behavioral Data

The U-Wilcoxon test revealed differences between ST and DT tasks in reaction time (Z = 4.457, *p* < 0.001), with the ST task being faster than the DT task (see mean values in Table 1). Regarding accuracy, the U-Wilcoxon test did not find differences between ST and DT tasks (Z = 0.416, *p* = 0.678).

### 3.2. ERP 

**Figure 2:**
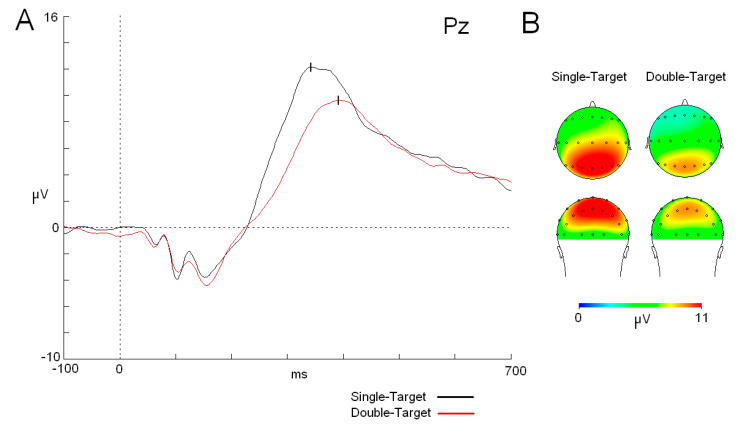
P3 component (modulations and topographic maps). (**A**) ERP traces for both tasks (single target (ST) (black trace) and double target (DT) (red trace)). Note that the ST task shows a shorter latency and higher amplitude for the P3 component compared to the DT task. (**B**) Maps (top and back views) of the P3 component in both tasks. Abbreviations: ms: milliseconds; µV: microvolts.

Regarding P3, a *t*-test showed that P3 latency reached the maximum amplitude earlier in the ST task than in the DT task (t_25_ = −6.043; *p* < 0.001) (see mean values in Table 1). On the other hand, there were differences in the P3 amplitude, which was lower in DT than in ST [F (1,25): 14.362; *p* < 0.001; ŋ^2^: 0.365] (Figure 2). The interaction between “cognitive load” × “anterior–posterior position” × “lateral–medial position” factors did not show a statistically significant difference between the two tasks due to a generalized increase in the amplitude in the ST task compared to that in the DT task in all homologous electrodes (*p* < 0.001) (Figure 2).

### 3.3. Alpha Band

**Figure 3:**
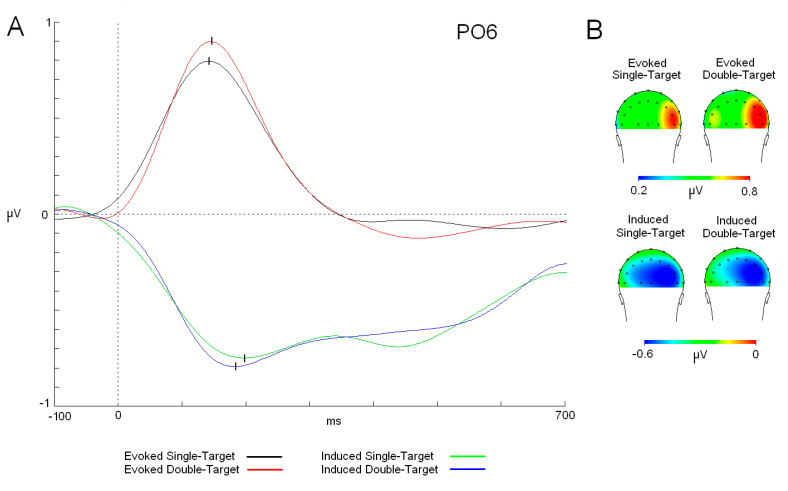
Alpha waves (modulations and topographic maps). (**A**) Evoked alpha modulation for both tasks (single target (ST) (black trace) and double target (DT) (red trace) and induced alpha activity (single target (ST) (green trace) and double target (DT) (blue trace). (**B**) Maps (back view) of the alpha distribution in both tasks. Abbreviations: ms: milliseconds; µV: microvolts.

**Figure 4:**
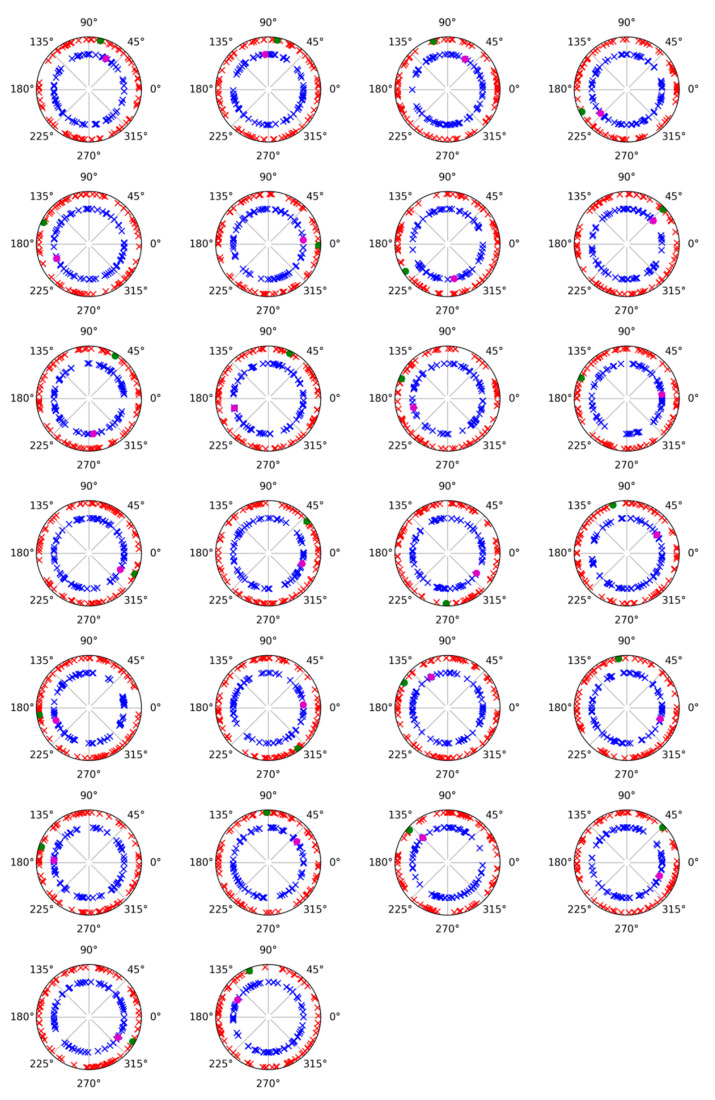
Polar plotting of phase values for evoked and induced alpha (8–13 Hz) activities and in each subject. The inner circle represents values for the ST task (red dot (evoked) and blue crosses (induced)) and the outer circle values for the DT task (green dot (evoked) and red crosses (induced)).

The analysis of latency between alpha activities showed that evoked response was faster than induced modulation [F (1,25): 8.408; *p* = 0.007; ŋ^2^: 0.252] (evoked: 149 ms; induced: 183 ms) (Figure 3). On the other hand, the cognitive load factor was not statistically significant as a main factor or in combination with the activity factor. In the amplitude analyses, ANOVA did not show any statistically significant difference due to the cognitive load or the activity factors in interaction with localization of the electrode factors (antero-posterior position or medial–lateral position). Moreover, evoked and induced modulations showed a similar distribution in the scalp, mainly evident in the right hemisphere, but with a different latency. Regarding the phase analysis, a phase plot showed that no relation was found between evoked and induced activities in either version of the task (ST or DT) (Figure 4).

## 4. Discussion

Behavioral data have shown that the reaction time variable of the DT task was slower than that of the ST task. This result is in accordance with previous studies in which the cognitive load of the task produced delays in reaction time [4,8,35,36]. Moreover, the percentage of accuracy showed almost a 100% value in both tasks, which indicates that no speed–accuracy trade-off was present in this study between tasks.

The cognitive load effect was not only relevant for the reaction time, but the P3 component showed a statistically significant delay in its latency for the more demanding experimental version (DT). This result supports previous studies where the delay of the P3 latency was modulated by the cognitive load of the task [1,2]. On the other hand, modulation of the P3 component by the cognitive load was not exclusively made over the latency, and amplitude was also significantly different with a decrease for the more demanding task (DT). Moreover, topographical analyses showed that there was no difference in P3 scalp distribution between both tasks. The difference observed here can be interpreted simply as a general reduction in the amplitude for all the derivations in the double-target task. Several authors have identified a similar result for this parameter in which the effect of the cognitive load was studied [2,35,37,38,39,40]. However, other studies have found no changes in P3 amplitude related to the cognitive load [4] or even an increase in the amplitude of this component related to cognitive load [36]. These contradictory results may be partly due to the different methodological approaches adopted across studies, as well as to the various dimensions encompassed by the variable “cognitive load”.

The effect of cognitive load on the amplitude of P3 has been interpreted in different ways. Several authors have proposed that the reduction in the amplitude is related to the amount of information transmitted during information processing. In other words, when the subject has a lower certainty about the stimuli (i.e., in a more demanding task), there is a reduction in the flow of information that is represented by a reduction in the amplitude of P3 [2]. Another interpretation has been formulated on the basis that if the task is repeated and becomes easier, mechanisms involved in the generation of P3 are more synchronized, and the amplitude becomes higher [41]. In any case, multiple processes have been related to the P3 component and not only related to resource allocation or working memory updating, as, for instance, referred to inhibition mechanisms [42]. Therefore, interpretation of the results for P3 has to be taken cautiously, and more research is needed to disentangle the precise meaning and relation of cognitive mechanisms involved in the P3 component.

Before analyzing the effect of the cognitive load over alpha modulations, it is relevant to highlight the delay of the induced activity in comparison to the evoked response. In previous studies, evoked and induced activity showed similar latencies (in some cases, almost identical), which led us to propose that both activities could represent linked mechanisms [16]. In particular, it was proposed in one of the studies that induced activity represents the reduction in “neural noise” in homologous areas where evoked responses took place [17]. This role could favor stimulus processing because of the reduction in potential competing alpha spectral content already present in the visual cortex. However, the confirmed delay in the current study for the induced activity suggests that it does not seem critical for this reduction to be in the same latency regarding the evoked response. Moreover, phase analyses have shown that induced activity in both tasks is randomly distributed in their polar values and suggest that no contribution between induced and evoked activities is present in the current study. This result has been observed in previous studies [19,20] and suggests that both activities (evoked and induced) are dissociable, although they could be partially related considering their similar topographical distribution.

Regarding the cognitive load effect on the evoked activity, statistical analysis did not show any modulation in the latency or amplitude parameters. A subtle increase in the amplitude was found in the DT task compared to that in the ST task (but the difference was not statistically significant). Considering that the evoked response could be considered as the spectral signature of the P1/N1 components [16,43], the present data would reflect an absence of modulation for these components due to the demanding level of the task. Previous studies have shown an increase or decrease in N1 amplitude related to the difficulty of the task [44,45]. The potential reason for these contradictory results could be due to the participation of different cognitive variables, such as allocation of attention and habituation [46]. Future studies are required to precisely disentangle the contributions of these mechanisms to spectral evoked modulation.

In the case of the induced activity as in the evoked response, amplitude and latency were not modulated by the cognitive load level of the tasks. In contrast to evoked activity, potential modulation of the induced response by cognitive load has not been studied before. In previous studies, some authors have demonstrated that cognitive load could take place in the early steps of stimulus processing (pP1 effect at 180 ms) [8,47]. However, with a similar latency for the induced alpha in the more demanding task (DT) (approximately 200 ms), no changes were seen with respect to the ST version.

A potential interpretation for the absence of modulation by cognitive load could be that the induced alpha decrement has reached the maximum value in both tasks (ground effect); therefore, no differences can be found between the two tasks. However, it seems reasonable that if the reaction time was shorter for the simple version of the task and P3 was also distinguished between both levels of cognitive load, alpha desynchronization could reflect some change related to this factor if it were related to it.

In our proposal that induced activity is related to a reduction in “neural noise” present in the visual cortex, the current results suggest that this reduction is not particularly affected by the cognitive mechanisms involved in cognitive load processing. However, some authors have found a stronger beta desynchronization during complex relative to simple sequences of movement executions [48]. It is necessary to emphasize at this point that some time–frequency techniques do not concretely analyze the non-phase modulation, which could affect the results observed [16]. The observed absence of a cognitive load effect over the induced modulation suggests that alpha desynchronization does not represent (at least evidently) the need for a higher number of neural resources to perform a more demanding version of the task. In other words, the decrement of induced alpha seems to represent a mechanism at a more sensory level facilitating the information processing and not determined by cognitive variables defined by the stimulus set (i.e., cognitive load). In any case, this interpretation of the results should be taken with caution. Other factors, such as task difficulty (as previously mentioned regarding a possible ground effect), as well as other aspects like individual variability observed in other types of paradigms [17], could influence the proposed conclusions. Considering that this is a preliminary study analyzing the potential effect of cognitive load on induced alpha activity, future studies using alternative approaches to cognitive load are necessary to confirm the explanatory hypothesis proposed in this manuscript.

Lastly, if the induced alpha activity does not seem to be related to the recruitment of neural sources to address the cognitive load of the task, the proposed compensatory mechanism in patients does not seem appropriate to justify larger amplitudes in alpha desynchronization, as has been suggested by a variety of authors. However, a higher decrement for alpha desynchronization has been commonly found in various studies and with different populations [19,23,49,50]. An alternative proposal to explain these results is that the decrement in alpha desynchronization is modulated by the motivational status of the experimental subject or group. In the case of pathological studies, patients are usually more motivated during the performance of the task, which could be the reason for a larger decrement in the induced alpha activity. However, this reduction would not be related to the involvement of more neural sources; instead, this reduction could be based on a general increase in arousal that aims to optimally reduce “neural noise” in the visual cortex to facilitate stimulus processing. Future studies in which motivational status may be controlled are needed to confirm this proposal.

## 5. Conclusions

In summary, evoked and induced alpha activities do not seem to be involved in the cognitive mechanisms related to the cognitive load of the task. Moreover, different parameters (latency and phase values) have shown that evoked and induced alpha responses are dissociable; however, at the same time, the similar distributions of the parameters suggest some sort of relation between these responses. Finally, the lack of modulation due to the cognitive load of induced alpha desynchronization does not support the hypothesis that this modulation could reflect compensatory mechanisms in pathological conditions.

## Figures and Tables

**Table 1:** Mean and standard deviation values of behavioral, P3 and alpha band activities.

Behavioral (Mean ± Standard Deviation)			
	**ST**	**DT**	**Wilcoxon Test (*p*-Value)**
Reaction Time	384 ± 57.09	447 ± 73.10	<0.001
Accuracy	99.55 ± 1.21	99.51 ± 1.33	0.678
**ERP**			
**Latency** (mean ± standard deviation)			
	**ST**	**DT**	***t*-Student (*p*-Value)**
P3	353 ± 44	405 ± 50	<0.001
**Alpha**			
**Latency** (mean ± standard deviation)			
	**ST**	**DT**	***t-*Student (*p*-Value)**
Evoked	146 ± 34	151 ± 28	1.000
Induced	168 ± 72	198 ± 95	0.197

Abbreviations: ST: single target; DT: double target; ERP: event-related potentials.

## Data Availability

The original data presented in the study are openly available in https://hdl.handle.net/11441/169348 (accessed on 25 February 2025).
